# Electrospun Membranes Loaded with Melanin Derived from Pecan Nutshell (*Carya illinoinensis*) Residues for Skin-Care Applications

**DOI:** 10.3390/membranes15020044

**Published:** 2025-02-03

**Authors:** Michell García-García, Jesús Salvador Jaime-Ferrer, Fernanda Nayeli Medrano-Lango, Elizabeth Quintana-Rodríguez, Tonatiu Campos-García, Erika Rodríguez-Sevilla, Domancar Orona-Tamayo

**Affiliations:** 1 CIATEC A.C., Centro de Innovación Aplicada en Tecnologías Competitivas, Omega 201, Industrial Delta, León C.P. 37545, Guanajuato, Mexico; mgarcia.picyt@ciatec.mx (M.G.-G.); equintana@ciatec.mx (E.Q.-R.); 2 Unidad Profesional Interdisciplinaria de Ingeniería Campus Guanajuato, Instituto Politécnico Nacional, Av. Mineral de Valenciana 200, Col. Fraccionamiento Industrial Puerto Interior, Silao de la Victoria C.P. 36275, Guanajuato, Mexico; fernay_medrano@yahoo.com; 3 CONACYT Research Fellow, CIATEJ. A.C., Centro de Investigación y Asistencia en Tecnología y Diseño del Estado de Jalisco, Av. de los Normalistas No. 800, Colinas de La Normal, Guadalajara C.P. 44270, Jalisco, Mexico; tocampos_pos@ciatej.edu.mx; 4 CIO. A.C., Centro de Investigaciones en Óptica, Loma del Bosque 105, Lomas del Campestre, León C.P. 37150, Guanajuato, Mexico; erika.rodriguez@cio.mx

**Keywords:** spinning fibers, molecular docking, bioactive compound, antioxidant activity, antimicrobial activity, antiaging effect

## Abstract

This study investigates the incorporation of melanin extracted from pecan nutshell residues into a polyacrylonitrile (PAN) matrix during the electrospinning of microfiber membranes. Melanin concentrations of 0.5, 2.0, and 5.0% *w*/*w* were incorporated to enhance the physicochemical and biological properties of the fibers. The melanin-loaded PAN fibers exhibited significant antioxidant activity against DPPH and ABTS radicals, with scavenging rates ranging from 46.58% to 62.77% and 41.02% to 82.36%, respectively, while unmodified PAN fibers showed no activity. Furthermore, the melanin-loaded membranes demonstrated antimicrobial effects. The membranes also exhibited an important enzyme inhibition activity against collagenase (37%), hyaluronidase (22%), tyrosinase (36%), and elastase (33%). Molecular docking studies reveal different potential amino acids of the active sites of aging enzymes that interact strongly with melanin pigment, particularly collagenase, followed by hyaluronidase, tyrosinase, and elastase. These results suggest that the novel melanin-loaded PAN membranes possess promising bioactive properties with potential applications in different skin-care applications.

## 1. Introduction

The skin is one of the human body’s largest and most versatile organs. In that regard, the skin is exposed continuously to different environmental factors (temperature variations, tobacco smoke, chemical pollutants, and radiation UV), and also, this organ has the ability to reduce negative environmental effects, decreasing mechanical damage, regulating critical physiological and biochemical processes, is sensitive to stimuli, regulates vitamin synthesis, promotes immune activity, and is sensitive to humidity [1,2]. Many of these stressful events trigger degenerative processes such as skin cancer, cellular aging, spots, and burns, as well as microbial infections [3]. Faced with these stressful events on the skin, people have sought solutions in cosmetic products made with ingredients of natural origin to feel better and to prevent damage, as well as aging, skin infections, and other aspects that can harm the skin. In that regard, in recent years, cosmetic industries, particularly in skin care, have been looking for new products with natural ingredients that help people who seek to have young and healthy skin to feel well.

The skin-care industry has shifted towards sustainability and the more efficient use of natural resources [4]. In this context, bioactive compounds obtained from agricultural biomasses have emerged as an innovative and environmentally friendly alternative for the formulation of cosmetic products. These compounds, derived from fruit and vegetable peels, herbs, coffee grounds, leaves, prunes, shells, and flower petals, benefit the skin and the environment [4,5,6]. Using natural compounds not only helps reduce the environmental impact by giving a second life to waste but also incorporates antioxidant, antiaging, antimicrobial, moisturizing, and regenerative properties that benefit skin health [6]. In addition, this practice supports the circular economy, aligning with the growing demands of conscious consumers looking for responsible and practical solutions in their personal care routines. Natural plant molecules remain particularly interesting for new development research to produce skin-care products with fewer side effects and rich sources of beneficial compounds for the skin.

Plant bioactive compounds can be used in new technologies that combine polymers and molecule bioactivities. One of these technologies is electrospinning, which can be used versatilely due to the use of biodegradable polymeric materials and uses electricity to produce functional biomaterials that can fuse bioactive molecules into their membranes with different target uses [7]. For example, in recent years, electrospinning technology has emerged as a promising avenue to overcome the limitations inherent in conventional skin-care approaches. The electrospinning technique has garnered significant attention due to its ability to fabricate nanofibrous or microfibrous membranes with exceptional properties, including high porosity, controlled drug release capabilities, and enhanced biocompatibility [8]. By combining the versatility of electrospinning with the potential of active compounds, researchers aim to develop materials that not only provide a physical barrier but also deliver active agents to the application site [9]. Active compounds such as antimicrobial and antioxidant agents, growth factors, antiaging compounds, and anti-inflammatory drugs can be incorporated into the electrospun fibers [10,11]. Several reviews have highlighted the potential advantages of electrospinning in skin-care applications. These reviews mainly summarize aspects of fiber technologies, including the synthesis methodologies [12,13,14,15], dressing composition [16,17], and nature of the materials employed, encompassing synthetic and natural polymers as well as self-responsive matter [18,19,20,21,22]. Furthermore, these reviews delved into the morphology, structure, and surface functionalization of the fibers [23,24], the bioactive constituents [25,26,27,28], the healing process of wounds and the therapeutic approach to reduce healing time, and antiaging effects [29,30].

Nowadays, the integration of active compounds derived from plant extracts [31,32], fruit peels [33,34], or agricultural by-product [35,36,37] with fibers has garnered significant attention in the field of skin-care applications. These approaches address environmental concerns associated with agricultural waste and the need for advanced materials with antimicrobial, antioxidant, and antiaging bioactivities. In this scenario, certain processing industries generate a significant amount of shell waste annually, most of which is disposed, leading to considerable environmental challenges [38,39]. In that sense, there is a comprehensive overview of various alternatives that have been investigated using electrospinning techniques, with the aim of imparting added value to shell wastes. These efforts represent a promising avenue for transforming environmental liabilities into valuable resources for skin-care applications.

The incorporation of pecan nuts (*Carya illinoinensis*) into the bakery and snack industry poses significant waste management challenges. This is primarily due to the substantial proportion of the original nut that is discarded during processing, which amounts to approximately 40 to 50% of its total mass. Most of this waste consists of the outer protective layer, or shell, that encases the edible kernel [40]. Pecan shells are also known for containing phenolic compounds (ranging from 32 to 117 mg GAE g^−1^), condensed tannins (with values between 130 and 357 mg CE g^−1^), and total anthocyanins (measuring 1 to 3 mg 3-glucoside cyanidin g^−1^) [41]. In addition to the previously listed compounds, melanin was recovered from the pecan shell with antioxidant and antifungal capacities [42], enhancing the value of an otherwise underutilized resource. Extracted melanin exhibits robust free-radical-scavenging properties, effectively neutralizing reactive oxygen species (ROS) and protecting cells from oxidative damage. This antioxidant activity plays a crucial role in mitigating the harmful effects of oxidative stress, which has been associated with various diseases and aging processes [43,44]. Ultraviolet (UV) radiation is a primary contributor to skin aging. Prolonged exposure to high levels of UV radiation induces the generation of ROS, leading to oxidative stress. This oxidative stress subsequently damages collagen and elastin fibers in the extracellular matrix (ECM), contributing significantly to the visible signs of skin aging. For example, the hyaluronidase enzyme regulates the hyaluronic acid degradation essential in skin hydration, while the tyrosinase enzyme is crucial for skin pigmentation; however, under oxidative stress, increased tyrosinase activity can produce skin hyperpigmentation [45]. Additionally, the ROS can increase the activity of the different aging enzymes, such as elastase, hyaluronidase, collagenase, and tyrosinase enzymes, that promote ECM degradation and the formation of photoaging signs. Therefore, bioactive compounds loaded into fibers could protect the skin from environmental pollutants to combat oxidative stress. Particularly, melanin extracts have demonstrated the ability to increase antioxidant enzyme activities, decrease lipid peroxidation, and potentially inhibit enzymes involved in cellular aging processes in a mice model [46].

In view of the bioactive properties of melanin outlined earlier, this new study aims to investigate the potential melanin-incorporated polyacrylonitrile (PAN) fibers as a novel skin-care material. Melanin extracted from Pecan nut (*C. illinoinensis*) shells was integrated into PAN fibers at varying concentrations (0.5%, 2.0%, and 5.0% *w*/*w*). This research encompasses a comprehensive evaluation of electrospinning parameters and extensive physico-chemical characterization of the resulting membranes. Furthermore, this study examines the degradation kinetics of the membranes and the release profile of melanin. This investigation extends to assessing the antioxidant properties of the fibers, their bacterial inhibition against *Escherichia coli*, *Staphylococcus aureus*, and *Pseudomonas aeruginosa* strains, and the capacity of melanin to inhibit aging-related enzymes including elastase, collagenase, tyrosinase, and hyaluronidase. Additionally, a molecular docking study was performed to determine the binding interactions between catalytic center amino acids and melanin pigment. This multifaceted approach aims to elucidate the potential applications of melanin-infused PAN membranes in advanced skin-care formulations.

## 2. Materials and Methods

### 2.1. Reagents

Polyacrylonitrile (PAN) (Mw 150,000), DPPH (1,1-diphenyl-2-picrylhydrazyl), ABTS (2,2′-azino-bis (3-ethylbenzothiazoline-6-sulfonic acid), tyrosinase enzyme (from mushroom, 1.14.18.1), 3,4-dihydroxy-L-phenylalanine (≥98%, CAS 59-92-7), collagenase enzyme (from *Clostridium histolyticum*, 3.4.24.3), Z-Gly-Pro-Gly-Gly-Pro-Ala-OH (collagenase substrate, CAS 13075-38-2), hyaluronidase enzyme (from Bovine testes, Type I-S, 3.2.1.35), Poly(β-glucuronic acid-[1→3]-β-N-acetylglucosamine-[1→4]) (alternating hyaluronic acid sodium salt from rooster comb, CAS 9067-32-7), elastase enzyme (from porcine pancreas, type IV, 3.4.21.36), and N-succinyl-L-alanyl-L-alanyl-L-alanine 4-nitroanilide (CAS 52299-14-6) were purchased from Sigma-Aldrich Chemical Reagent Co., Ltd. (Saint Louis, MO, USA). N,N-dimethylformamide (N,N-DMF, 99.8%), chloroform (CHCl_3_, >99.5%), ammonium hydroxide (NH_4_OH, 28.0–30.0%), and hydrochloric acid (HCl, 36.5–38.0%) were purchased from Karal^TM^ (Guanajuato, Mexico). *Escherichia coli*, *Staphylococcus aureus*, and *Pseudomonas aeruginosa* were obtained from the American Type Culture Collection (ATCC, Manassas, VA, USA).

### 2.2. Melanin Extraction

The method for obtaining melanin from the pecan shell was performed following the method described by García et al. [42]. Briefly, the shells were washed with running water by immersing them in water at 25 °C (1:10) and adding NH_3_•H_2_O to adjust the pH to 10.5 (10 g/100 g) to a final concentration of 2 g/100 g. They were then incubated at room temperature (24 h). Following incubation, the mixture was filtered, and the filtrate was adjusted to a pH of 2.5 with 2 mol L^−1^ HCl. The acidified solution was then filtered again to obtain raw melanin.

### 2.3. Melanin Purification

The raw melanin was hydrolyzed with 7 mol L^−1^ HCl for 6 h at 100 °C; the residues that were not hydrolyzed were centrifuged at 10,000 rpm for 10 min and resuspended at 10% NH_3_•H_2_O. Sequentially, three washes were made with chloroform and ethyl acetate. The aqueous phase was acidified with 1 mol L^−1^ HCl, and the filtered residue was washed with water. The residue (solid matter) was again dissolved in 1 mol L^−1^ NH_3_•H_2_O and filtered. The supernatant was acidified with 1 mol L^−1^ HCl, and the filtered residue was washed with H_2_O to obtain the purified melanin.

### 2.4. Fiber Preparation

Solutions for electrospinning were prepared by solubilizing polyacrylonitrile (PAN) in N,N-dimethylformamide (N,N-DMF) in a ratio of 12:88% (*w*/*w*) [47]. The mixtures were agitated for 24 h. The solutions were prepared by adding the powdered and sieved melanin powder (SIEVE TEST No. 230; 63 µm), in concentrations of 0.5% (PM0.5), 2.0% (PM2.0), and 5.0% (PM5.0) (*w*/*w*). Membranes were fabricated using the NE100 Single Nozzle Electrospinning/Spraying Machine (INOVENSO Ltd., Istanbul, Turkey). A total of 6 mL of each polymeric solution was injected at a flow rate of 0.1 mL h^−1^. The solution was then projected onto a rotating drum covered with an aluminum film, spinning at 300 rpm. The distance between the nozzle and the collector was 17 cm, and a voltage of 15 kV was applied.

### 2.5. Fiber Characterization

#### 2.5.1. Morphology Analysis

Fiber morphologies were analyzed by a field-emitting scanning electron microscope (FESEM) (Hitachi S-4800, Tokyo, Japan) at an acceleration voltage of 2 kV and a current flow of 10 μA. For characterization, a small part of each sample was coated with 9 nm gold/palladium to reduce the loading effect before imaging (Polaron equipment, SEM E5100 coating unit, Kontron AG, Switzerland). The software ImageJ 1.54 (National Institutes of Health, Bethesda, MD, USA) was used to measure the diameter of the fiber.

#### 2.5.2. Chemical Composition Analysis

To identify the functional groups and chemical bonds present in PAN–melanin fibers, Fourier transform infrared spectroscopy (FTIR) analysis was conducted using a Nicolet iS10 instrument from Thermo Scientific (Waltham, MA, USA) equipped with an attenuated total reflectance (ATR) diamond accessory. The analysis followed the guidelines outlined in ASTM E573-01(2013) [48] and covered a range of 4000 cm^−1^ to 400 cm^−1^.

#### 2.5.3. Wettability Analysis

To evaluate the water absorption capability of each sample, the 1 cm^2^ membranes were immersed in phosphate buffer (PBS) (37 °C) or water (room temperature) for 24 h, and both the dry $\left(W_{d}\right)$ and wet $\left(W_{s}\right)$ weight of the membranes were recorded.

Subsequently, the water uptake was determined using Equation (1) [49]:

(1) $$Water\;uptake=\frac{W_{s}-W_{d}}{W_{d}}\times 100$$

The contact angle was determined following the methodology proposed by Ruiz-Rocha et al. [50], using the OCA 15EC equipment (Dataphysic, Filderstadt, Germany). Ten reference points were selected to examine the interaction between water and both ground melanin and electrospun fibers.

#### 2.5.4. Mechanical Characterization

In compliance with the ASTM-882 standard governing thin plastic films, the Young’s modulus, tensile strength, and elongation percentage of the membranes will be assessed using 100 mm × 10 mm specimens [51], at a rate of 10 mm min^−1^, as determined by Equation (2) and executed using the INSTRON 5565 (Canton, MA, USA). Polyacrylonitrile (PAN) is a highly versatile polymer due to its high carbon content. PAN exists in semi-crystalline and amorphous polymer forms and has a degree of crystallinity of 35.05%. The physical, chemical, electrical, mechanical, and thermal properties strongly depend on the polymer’s crystalline structures [52,53]. Crystalline solids are inherently anisotropic, whereas amorphous solids are isotropic. Therefore, being an anisotropic material [54], ten specimens, five normal and five parallel with the principal axis of anisotropy, were tested from each sample. The rate of grip separation will be determined for the purpose of these test methods based on the initial strain rate as follows:

(2) A=B×C

where A denotes the separation speed in mm min^−1^, B represents the initial distance between the jaws in mm, and C signifies the initial deformation rate obtained from the standard and is utilized as specified by the standard for speed calculation.

#### 2.5.5. Liberation of Melanin

The methodology of Kalantary et al. [55] was used, with some modifications. A total of 1 mg of the membrane was weighed, subsequently immersed in 3 mL of PBS pH 7.4, and finally incubated with shaking at 180 rpm and 37 °C. Samples of 1 mL were taken every 2 h, for a total period of 10 h, and the same volume of solution was applied to the incubation solution. The samples were read on the Evolution 300 from Thermo Scientific (Madison, WI, USA) equipment at 220 nm, determining the concentration using a previously created calibration curve.

### 2.6. Fiber Bioactivities

#### 2.6.1. Antioxidant Activity Against DPPH

The DPPH radical scavenging assay was conducted following a modified version of the methodologies described by Orona-Tamayo et al. [56] and Kalantary et al. [55]. A DPPH radical solution of 0.000180 mol L^−1^ was prepared in 80/100 mL of methanol. For sample preparation, 1 mg of each fiber was dissolved in 1 mL of N,N-DMF solution, maintaining a 1:1 ratio of the sample to the antioxidant in a 96-well plate. The plate was then placed in darkness and incubated at 37 °C for 30 min. After incubation, the absorbance was measured at 517 nm using the Multiskan SkyHigh microplate spectrophotometer from Thermo Fisher Scientific (Waltham, MA, USA). The results were computed using Equation (3).

(3) $$RSA(\%)=\left(1-\frac{A_{i}-A_{j}}{A_{c}}\right)\times 100$$

where $A_{c}\;$is the absorbance of the radical without the sample, $A_{i}$ is the absorbance of the tested sample, and $A_{j}$ is the absorbance of the blank group (n=3).

#### 2.6.2. Antioxidant Activity Against ABTS

The experiment followed the methodology proposed by Orona-Tamayo et al. [56], albeit with some modifications. Initially, an ABTS solution (0.007 mol L^−1^, 3 mL) and APS (0.00245 mol L^−1^, 15 mL) were dissolved in distilled water and mixed in darkness at 25 °C for 16 h. The resulting blue/green ABTS radical solution was then freshly prepared and adjusted with 100% methanol to achieve an absorbance of 0.700 ± 0.02, measured at 734 nm. Sample preparation involved dissolving 1 mg of each membrane in 1 mL of N,N-DMF solution, maintaining a 1:1 ratio of sample to antioxidant. Subsequently, the plate was incubated at room temperature for 6 min, followed by an absorbance measurement at 734 nm using the Multiskan SkyHigh microplate spectrophotometer from Thermo Fisher Scientific (Waltham, MA, USA). A decrease in absorbance indicates higher antioxidant activity. The results were calculated using Equation (3).

#### 2.6.3. Antimicrobial Activity

The antimicrobial efficacy of the electrospun fibers was evaluated using the disk diffusion method, performed in triplicate. Circular samples with a diameter of 5 mm were prepared from each fiber. *E. coli* was used (gram-negative, enteropathogenic) along with two opportunistic bacteria, *S. aureus* (gram-positive skin bacterium) and *P. aeruginosa* (a gram-negative respiratory tract pathogen). The strains were activated by inoculation on nutrient agar and then reseeded in nutrient broth. As a positive control, gentamicin and ampicillin (0.1 mg mL^−1^) embedded in filter paper (diameter of 5.0 mm; thickness of 1.0 mm) were used. Therefore, 100 µL of each strain was inoculated and measured at 600 nm (${OD}_{600}$); samples were read on the Evolution 300 from Thermo Scientific (Madison, WI, USA) to estimate cell density or bacterial growth in liquid culture. The plates were then incubated at 37 °C for 24 h. Antimicrobial capacity was reported by the inhibition halo (mm) generated for each sample on the plates.

### 2.7. Antiaging Effect

The enzymatic activities were performed based on the methodology of Aguilar-Toalá and Liceaga [57], with some modifications, using tyrosinase, collagenase, elastase, and hyaluronidase enzymes. Samples consisted of melanin dissolved in saline phosphate buffer (0.5 mM) at pH 7.2 in the different concentrations of solution (0.5%, 2.0%, and 5.0% *w*/*w*) in which the membranes were made.

#### 2.7.1. Elastase Inhibition Assay

The assay was performed in a 96-well microplate format, and 5 µL of the melanin solution was combined with 5 µL of 10 mM substrate *N*-succinyl-Ala-Ala-Pro-Val-*p*-nitroanilide (S4760, Sigma-Aldrich) dissolved in DMSO. This mixture was incubated for 15 min at 37 °C. Afterward, 50 μL of type IV porcine pancreatic elastase (10 mU in 100 mM Tris-HCl buffer; pH 8.0) was added and pre-incubated 5 min at 37 °C, after this time, 140 µL of 100 mM Tris-HCl buffer (pH 8.0) was added, and the mixture was incubated at 37 °C. A change in absorbance was recorded every 10 min for 60 min with measurements at 405 nm using a microplate spectrophotometer (Multiskan SkyHigh, Thermo Fisher Scientific). The percentage of inhibition was calculated using Equation (4), where ${OD}_{control}$ and ${OD}_{sample}$ represent the optical density of the control and samples, respectively [57].

(4) $$Enzymatic\;inhibition\;\left(\%\right)=\frac{{OD}_{control}-{OD}_{sample}}{{OD}_{control}}\times 100$$

#### 2.7.2. Tyrosinase Inhibition Assay

The tyrosinase inhibition assay was conducted in a 96-well microplate format. The procedure involved combining 5 µL of melanin samples with 5 µL of 10 mM 3,4-dihydroxy-L-phenylalanine (L-DOPA; D9628, Sigma-Aldrich) substrate dissolved in 0.5 mM phosphate-buffered saline (PBS; pH 7.2). This mixture was incubated for 15 min at 30 °C. Subsequently, 50 μL of pre-incubated mushroom tyrosinase (75 mU in 0.5 mM PBS; pH 7.2) was added and incubated for an additional 5 min at 37 °C. Following this incubation, 140 µL of 0.5 mM PBS (pH 7.2) was added to each well. Absorbance measurements were recorded at 405 nm every 10 min for a total duration of 60 min using a Multiskan SkyHigh microplate spectrophotometer (Thermo Fisher Scientific). The percentage of tyrosinase inhibition was calculated using Equation (4), as described in the literature [57].

#### 2.7.3. Collagenase Inhibition Assay

The inhibition enzyme assay was performed in a 96-well microplate format, and 5 µL of the melanin samples were combined with 5 µL of 1 mM substrate Z-Gly-Pro-Gly-Gly-Pro-Ala-OH (27673, Sigma-Aldrich) dissolved in 50 mM Tris-HCl buffer; pH 7.5. This mixture was incubated for 15 min at 30 °C. Afterward, 50 μL of pre-incubated collagenase from *Clostridium histolyticum* (5 min, 30 °C, and 50 mU; 50 mM Tris-HCl buffer; pH 7.5) was added. After this time, 140 µL of 50 mM Tris-HCl, pH 7.5, was added. A change in absorbance was recorded every 10 min for 60 min with measurements at 340 nm using a microplate spectrophotometer (Multiskan SkyHigh, Thermo Fisher Scientific). The percentage of inhibition was calculated using Equation (4) [57].

#### 2.7.4. Hyaluronidase Inhibition Assay

The assay was performed using a 96-well microplate format, for which 5 µL of the melanin samples were combined with 5 µL of 1.4 mg mL^−1^ substrate hyaluronic acid sodium salt (H5388, Sigma-Aldrich) dissolved in acetate buffer (50 mM; pH 4.5). This mixture was incubated for 15 min at 37 °C. Afterward, 50 μL of pre-incubated hyaluronidase (5 min; 37 °C) in acetate buffer (50 mM; pH 4.5) was added. After this time, 140 µL of 50 mM acetate buffer, pH 4.5, was added. A change in absorbance was recorded every 10 min for 60 min with measurements at 550 nm using a microplate spectrophotometer (Multiskan SkyHigh, Thermo Fisher Scientific). The percentage of inhibition was calculated using Equation (4) [57].

### 2.8. Docking In Silico of Each Tested Enzymes with Melanin Results

For this analysis, the core structure of melanin was fetched from the PubChem database (https://pubchem.ncbi.nlm.nih.gov/; accessed on 29 October 2024). The 3D crystal structures of the peptidase domain of collagenase G from *Clostridium histolyticum* (PDB ID 7Z5U) [58], human hyaluronidase 1 (PDB ID 2PE4) [59], human neutrophil elastase (PDB ID 3Q77) [60], and tyrosinase from *Bacillus megaterium* (PDB ID 6EI4) [61] were downloaded from the Protein Data Bank (PDB) at the Research Collaboratory for Structural Bioinformatics (RCSB) (https://www.rcsb.org/; accessed on 29 October 2024) [62]. Molecular docking simulations were conducted using the “Ligand Docker” module within the CHARMM-GUI web interface (https://www.charmm-gui.org/; accessed on 29 October 2024) [63]. The AutoDock Vina algorithm, which employs the Lamarckian Genetic Algorithm and Empirical Free Energy Scoring Function [64], was used for docking. Protein–ligand interactions within a 4 Å radius were analyzed and visualized using the web-based tool PLIP (Protein–Ligand Interaction Profiler) [65]. Images for the figures were generated using the molecular visualization software PyMOL (Version PyMOL(TM) 2.5.8) [66]. Simultaneously, experimentally crystallized inhibitors were re-docked under identical conditions to facilitate a comparative analysis of binding poses and energies.

### 2.9. Statistical Analysis

ANOVA (Analysis of Variance) was conducted following a multivariate test using Tukey’s test for post hoc analysis. All graphs and analyses were performed using RStudio 2024.12.0+467 software for Windows.

## 3. Results and Discussions

### 3.1. Membrane Elaboration

**Figure 1:**
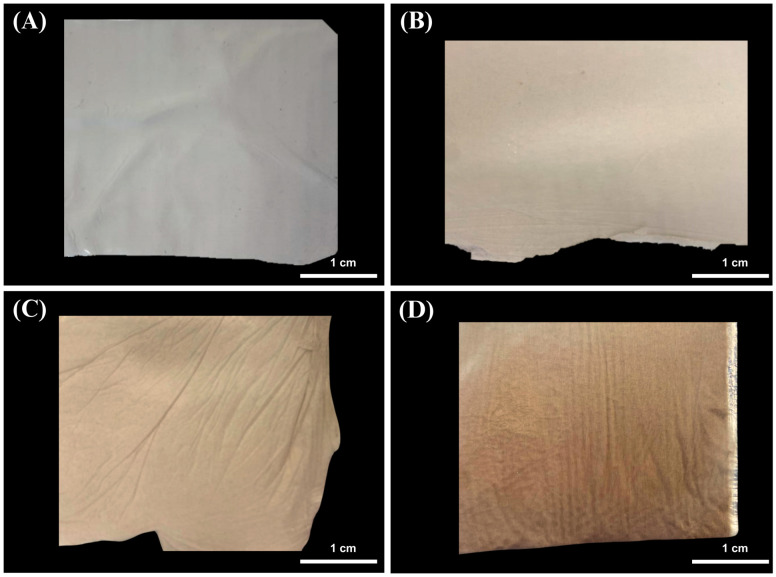
Fibers composed of polyacrylonitrile (PAN) and melanin pigment. (**A**) Control (PAN), (**B**) PAN + 0.5% of melanin (PM0.5), (**C**) PAN + 2.0% of melanin (PM2.0), and (**D**) PAN + 5.0% of melanin (PM5.0).

Four distinct membranes were successfully fabricated using varying melanin concentrations to obtain the physical fibers (Figure 1). The electrospinning parameters were maintained constant for each membrane, while non-controllable parameters, such as temperature and humidity, were closely monitored. The resulting membranes exhibited a visible color transition turning from white to brown, with the shade intensifying proportionally to the concentration (Figure 1). This observation aligns with previous studies on polycaprolactone (PCL) and polyurethane (PUR) fibers loaded with different melanin concentrations, which similarly demonstrated an increase in color intensity as melanin content increased, resulting in progressively darker fibers [67,68]. Our fabricated materials displayed comparable color changes, indicating successful incorporation of melanin into the fiber structure.

### 3.2. Light and Scanning Electron Microscopy

**Figure 2:**
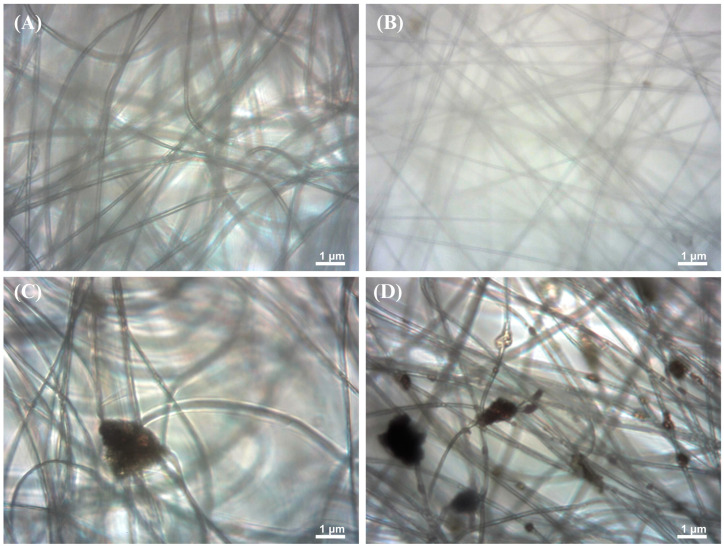
Light microscopy images of fiber membranes composed by PAN and melanin pigment. (**A**) Control (PAN), (**B**) PAN + 0.5% of melanin (PM0.5), (**C**) PAN + 2.0% of melanin (PM2.0), and (**D**) PAN + 5.0% of melanin (PM5.0).

The light microscopy analysis of fiber morphology revealed distinct characteristics across different melanin concentrations (Figure 2A). Control PAN fibers without melanin exhibited uniform and clean extension, free from dark particles or inclusions, indicating a pure and homogeneous structure. PAN fibers with 0.5% melanin (PM0.5, Figure 2B) appeared like the control, with a slight difference in density and distribution. These fibers remained mostly clean with minimal dispersed particles, suggesting initial melanin incorporation into the fiber matrix.

PAN fibers with 2% melanin concentration (PM2.0, Figure 2C) showed the emergence of dark particles distributed along the fibrous structure. These inclusions indicated a higher presence of melanin within the fibers, resulting in a more complex and heterogeneous texture compared to lower concentrations. The highest melanin concentration of 5% (PM5.0, Figure 2D) revealed a considerable number of dark particles and aggregates dispersed among the fibers. This micrograph displayed a densely packed network of fibers with visible melanin inclusions, potentially influencing the fibers’ mechanical and functional properties. This progressive series of images clearly demonstrates how increasing melanin concentrations alter the morphology and composition of polyacrylonitrile fibers, providing valuable insights into the integration of bioactive compounds in fibrous materials.

**Figure 3:**
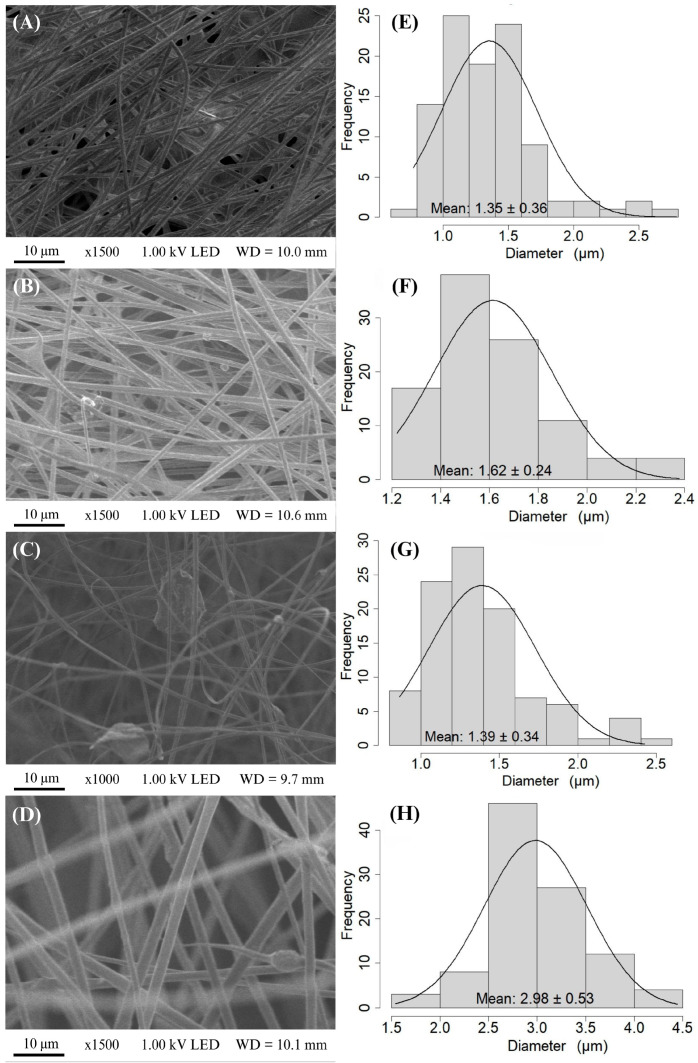
SEM micrographs of fiber composed by PAN and melanin pigment. (**A**) Control (PAN), (**B**) PAN + 0.5% of melanin (PM0.5), (**C**) PAN + 2.0% of melanin (PM2.0), and (**D**) PAN + 5.0% of melanin (PM5.0) and diameter size distribution histogram of samples labeled as (**E**–**H**), respectively, n=100.

The electrospun fibers in the fabricated membranes were characterized using scanning electron microscopy (SEM) micrographs. The micrographs of the PAN (Figure 3) and PAN–melanin membranes at different concentrations (Figure 3B–D) show the alignment of fibers due to the equipment configuration and the type of collector used.

The fibers exhibit surface roughness in their morphological structure. Additionally, the presence of particles is notable in the membranes of the PAN + 0.5% melanin (PM0.5), PAN + 2.0% melanin (PM2.0), and PAN + 5.0% melanin (PM5.0) treatments. The number of particles increases as the melanin concentration rises, along with an increase in the fiber diameter. The electrospun PAN fibers have a diameter around 1.35 µm ± 0.36 µm, while fibers containing melanin range in diameter from 1.39 µm ± 0.34 µm to 2.98 µm ± 0.53 µm. As the concentration of melanin increases in the PAN fibers, the diameter of the fibers also increases.

This phenomenon can be attributed to several interconnected factors associated with the electrospinning process and the intrinsic properties of the material. As the melanin concentration increases, the viscosity of the polymer solution correspondingly rises. A more viscous solution is less prone to stretching during the electrospinning process, leading to the formation of thicker fibers [69,70]. Additionally, highly concentrated polymer solutions experience accelerated solvent vaporization, which restricts polymer extension and accelerates compaction, resulting in the formation of thicker fibers [71]. Melanin can alter the electrical conductivity of the solution. These changes in conductivity can affect the stability of the electrospinning jet and the stretching forces applied to the fibers, often resulting in thicker fibers. Therefore, a decrease in conductivity, likely caused by the melanin, results in fibers with lower conductivity and greater thickness [69]. Finally, the mechanical properties of the polymer solution, such as elasticity and plasticity, are affected by the concentration of melanin. These changes impact how the solution behaves under the electric field during electrospinning, leading to variations in fiber thickness.

### 3.3. Chemical Properties of Fibers

**Figure 4:**
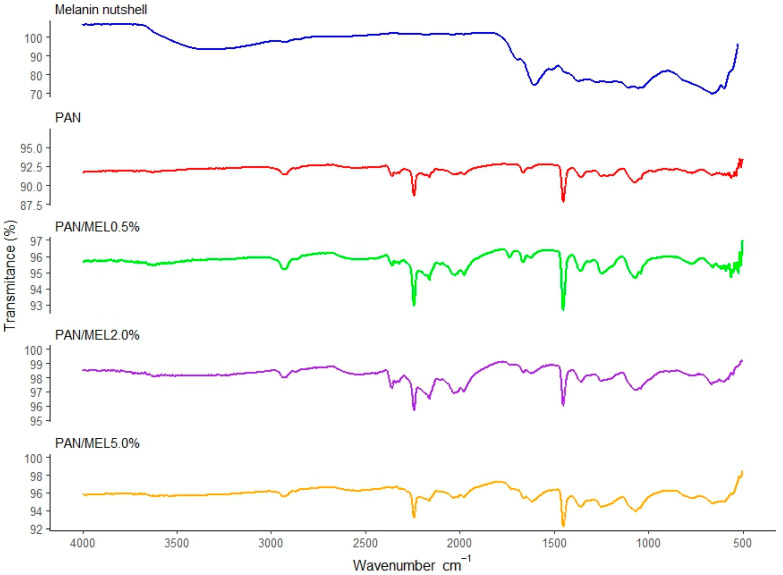
Infrared spectra (FTIR) within a wavenumber range of 4000 cm^−1^ to 500 cm^−1^ of melanin from nutshell and membranes obtained with melanin pigment, PAN control, PAN + 0.5% of melanin (PM0.5), PAN + 2.0% of melanin (PM2.0), and PAN + 5.0% of melanin (PM5.0) fibers.

Figure 4 shows the infrared spectra (FTIR) of membranes obtained with the PAN formulations PAN + 0.5% melanin (PM0.5), PAN + 2.0% melanin (PM2.0), and PAN + 5.0% melanin (PM5.0). The FTIR spectra of the PAN fibers shows characteristic peaks at 2931 cm^−1^ (γ CH_2_), 2242 cm^−1^ (γ C≡N), and 1664 cm^−1^ (γ C=O) of the residual N,N-DMF and 1453 cm^−1^ (δ CH_2_), where γ represents a stretching vibration and δ denotes a bending vibration. Additionally, it is possible to observe the peak located at 1249 cm^−1^ related to the methine vibration, and the peak at 1071 cm^−1^ corresponds to the C-H interaction [72,73]. The absorption peaks of these characteristics are still present in the fibers added with melanin, indicating that melanin does not destroy or weaken the original molecular structure of PAN fibers and that some characteristics of the original PAN fibers are preserved, highly comparable peaks to those obtained by Naragund and Panda [74] and Yao et al. [75]. The wavenumber region between 1000 cm^−1^ and 500 cm^−1^ exhibits a significant amount of noise, which prevents the identification of characteristic peaks in this specific region.

Furthermore, Figure 4 shows the comparison of the infrared spectra obtained from melanin derived from walnut shells [42] and electrospun PAN fibers, along with their infusions at different melanin concentrations (PM2.0 and PM5.0). The melanin extracted from pecan shells [42] is an allomelanin that exhibits characteristic peaks at 3352 cm^−1^ associated with the hydroxyl group and at 1106 cm^−1^ and 1056 cm^−1^ attributed to C-O bonds, and in the spectral range between 700 cm^−1^ and 600 cm^−1^, C-S bonds are observed. However, when melanin is infused into the polymeric fibers of polyacrylonitrile, these characteristic peaks are not present. The absence of the characteristic melanin peaks when infused into polyacrylonitrile polymer fibers can occur for several reasons: (i) through a chemical interaction, melanin may chemically interact with PAN, which could alter the structure of melanin or change its spectroscopic properties; (ii) in the matrix environment, melanin could be dispersed differently or adopt a different structure when incorporated into the PAN matrix, which could affect the observation of its characteristic peaks; (iii) with regard to the dispersion state, the way melanin is dispersed in PAN fibers can influence the accessibility of functional groups to interact with infrared radiation; (iv) with regard to structural modifications, during the infusion process into PAN fibers, melanin could undergo structural or conformational changes that affect the position or intensity of its spectral bands [76,77,78].

### 3.4. Wettability Results

**Figure 5:**
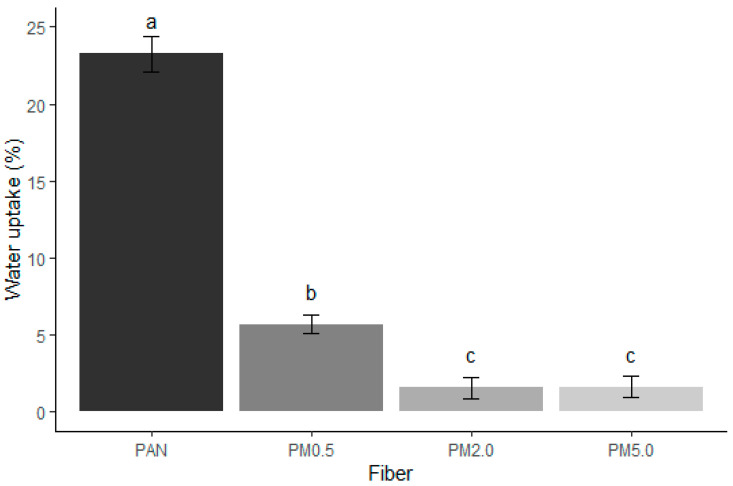
Degree of swelling of electrospun membranes composed of PAN and melanin pigment. Control (PAN), PAN + 0.5% of melanin (PM0.5), PAN + 2.0% of melanin (PM2.0), and PAN + 5.0% of melanin (PM5.0). Bars represent means ± SE (n=3) of the water uptake in %. Different letters indicate significant differences between all factor combinations (univariate ANOVA after post hoc Tukey test, *p* < 0.05).

Figure 5 shows that the incorporation of melanin into the PAN membranes significantly reduces their water uptake capacity. The recorded absorption percentages were 23.25 ± 1.16% for control PAN fibers, 5.69 ± 0.57% for PM0.5 fibers, 1.58 ± 0.68% for PM2.0 fibers, and 1.63 ± 0.70% for PM5.0 fibers. PAN is inherently hydrophobic. However, the lone-pair orbital located on nitrogen and oriented 180° relative to the C≡N bond is associated with hydrogen bonding that could interact with water [79]. The overall structure of PAN and the arrangement of its molecules do not favor an affinity for water. This characteristic makes it resistant to water absorption and other polar solvents. The higher the melanin concentration, the lower the degree of swelling, with PM2.0 and PM5.0 fibers showing the least water uptake. This suggests that melanin effectively reduces the hydrophilicity of the PAN fibers, making them less prone to swelling in the presence of water.

The incorporation of melanin into PAN fibers reduces their hydrophilicity and swelling in the presence of water due to the hydrophobic nature of melanin, the creation of a denser and less porous fiber structure, increased cross-linking density, and the formation of a hydrophobic phase that acts as a barrier to water absorption. Surfaces with a water contact angle below 60° are considered hydrophilic, while those with contact angles above 90° are considered hydrophobic. When the contact angle exceeds 120°, the surface is classified as super-hydrophobic [80].

**Figure 6:**
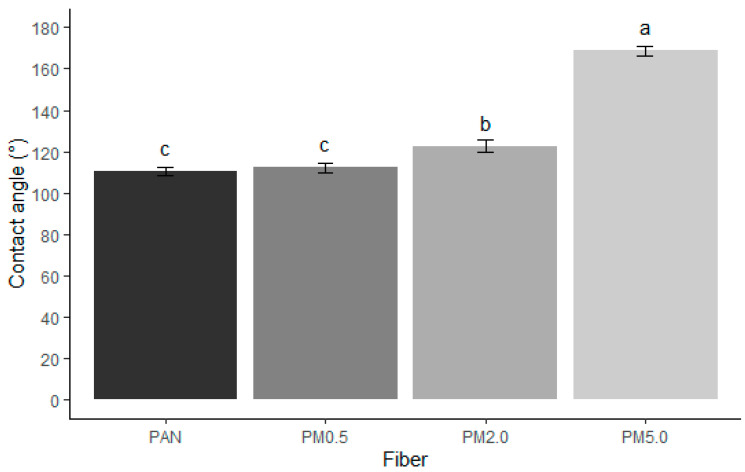
Contact angle of electrospun membranes composed of PAN and melanin pigment. Control (PAN), PAN + 0.5% of melanin (PM0.5), PAN + 2.0% of melanin (PM2.0), and PAN + 5.0% of melanin (PM5.0). Bars represent means ± SE (n=10) of the contact angle in degrees. Different letters indicate significant differences between all factor combinations (univariate ANOVA after post hoc Tukey test, *p* < 0.05).

PAN fibers exhibit hydrophobic behavior, with a contact angle of 110.59° ± 1.95°, which constitute very similar results to those reported by Sanchaniya and Kanukuntla [80] for a 12% (*w*/*w*) PAN concentration. It is possible to observe an increase in the hydrophobicity of the fibers as the concentration of melanin that is present increases. Melanin is a hydrophobic compound (right contact angle 136.16° ± 0.77°; left contact angle 136.00° ± 0.96°) [42], which, when combined with PAN in high concentrations, considerably increases its hydrophobicity (Figure 6).

### 3.5. Fibers Degradation

**Figure 7:**
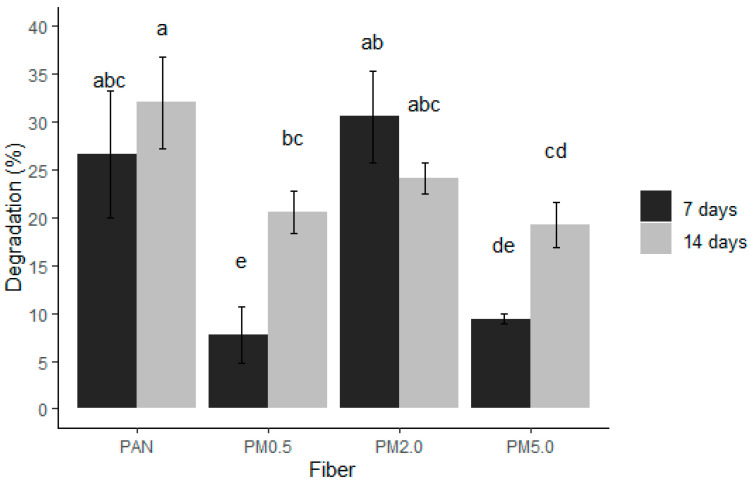
PAN and PAN–melanin fiber degradations for two weeks. Control (PAN), PAN + 0.5% of melanin (PM0.5), PAN + 2.0% of melanin (PM2.0), and PAN + 5.0% of melanin (PM5.0). Bars represent means ± SE (n=3) of the fiber degradation in %. Different letters indicate significant differences between all factor combinations (univariate ANOVA after post hoc Tukey test, *p* < 0.05).

The degradation rates of the control PAN and PAN membranes loaded with melanin were monitored over 7 and 14 days (Figure 7). The data show distinct differences in degradation percentages across various melanin concentrations and time periods. The control PAN membranes exhibited a degradation percentage of 26.6 ± 6.6% after 7 days, increasing to 32.02 ± 4.8% after 14 days. For the PAN + 0.5% melanin (PM0.5) membrane, the degradation percentage was notably lower at 7.7 ± 2.9% after 7 days and rising to 20.6 ± 2.2% after 14 days. The PAN + 2.0% melanin (PM2.0) membrane showed a degradation of 30.5 ± 4.8% after 7 days and 24.0 ± 1.6% after 14 days. The PAN + 5.0% melanin (PM5.0) membrane had the lowest degradation rates, around 9.4 ± 0.5% after 7 days and 19.3 ± 2.3% after 14 days. Melanin’s inherent stability and resistance to environmental degradation likely contribute to the lower degradation rates observed in PAN–melanin membranes. As the concentration of melanin increases, the protective effect becomes more pronounced, resulting in a lower degree of fiber degradation. Also, the incorporation of melanin can enhance the structural stability of the membranes. By increasing cross-linking density and forming a more robust polymer matrix, melanin reduces the susceptibility of the fibers to degradation processes. Finally, melanin imparts hydrophobic properties to the PAN fibers, reducing water absorption and thus limiting hydrolytic degradation. The dominant characteristic of the PAN molecule is the presence of strong polar nitrile groups. The CN groups have a wide range of possibilities for interacting with their surroundings. The high dipole moment can cause strong attraction or repulsion (depending on orientation) of other molecules or substituents in molecules that also have a high dipole moment [79]. PAN differs in many ways from common commercial polymers. Its typical properties include hardness, rigidity, and resistance to most solvents and chemicals. PAN only dissolves in (i) aprotic polar organic solvents such as dimethylformamide, dimethylacetamide, dimethyl sulfoxide, sulfolane, ethylene carbonate, and N-methylpyrrolidone; (ii) concentrated sulfuric acid and nitric acid; and (iii) concentrated aqueous solutions of certain inorganic salts, such as lithium bromide, sodium thiocyanate, and zinc chloride. Acrylonitrile copolymers are often soluble in less polar organic solvents, such as dioxane, tetrahydrofuran, chlorobenzene, cyclohexanone, and acetone [81], which makes them highly resistant. Then, less water uptake means slower breakdown of the polymer chains, leading to lower degradation rates.

**Figure 8:**
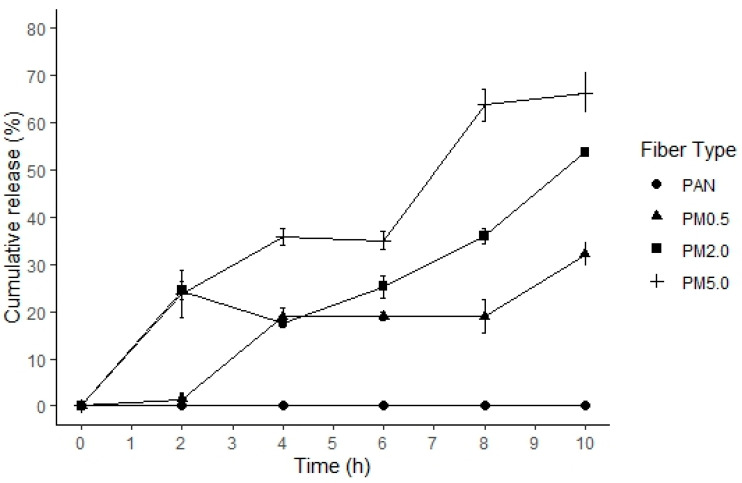
The in vitro release profiles of melanin from different PAN and PAN–melanin fibers over time. Control (PAN), PAN + 0.5% of melanin (PM0.5), PAN + 2.0% of melanin (PM2.0), and PAN + 5.0% of melanin (PM5.0). Points represent means ± SE (n=3) of the melanin liberation from fibers.

Figure 8 shows the melanin release kinetics that were expressed as a percentage over the time for control PAN and loaded fibers with different melanin concentrations. The release of the melanin from the PAN fibers occurred gradually during the 10 h kinetics assessment, with the PAN membrane with 5.0% of melanin (PM5.0) exhibiting a higher percentage of release (66.26 ± 4.29%) compared to the control PAN membranes. However, during the first two hours, the PAN–melanin membranes at 2.0 (PM2.0) and 5.0% (PM5.0) have the highest melanin release (23.66 ± 5.04% and 24.42 ± 1.91%, respectively), while the PAN–melanin membrane at 0.5% (PM0.5) had the maximum release from 2 to 4 h. All PAN–melanin membranes showed a constant release of melanin from the point of maximum release. Gradually, a more significant release is produced by PAN membranes with higher melanin contents. This results from the increased presence of melanin within the fiber matrix, which can disperse with greater ease. Higher melanin content may disrupt the fiber matrix, facilitating easier diffusion of melanin particles.

### 3.6. Mechanical Properties of the Membrane

**Figure 9:**
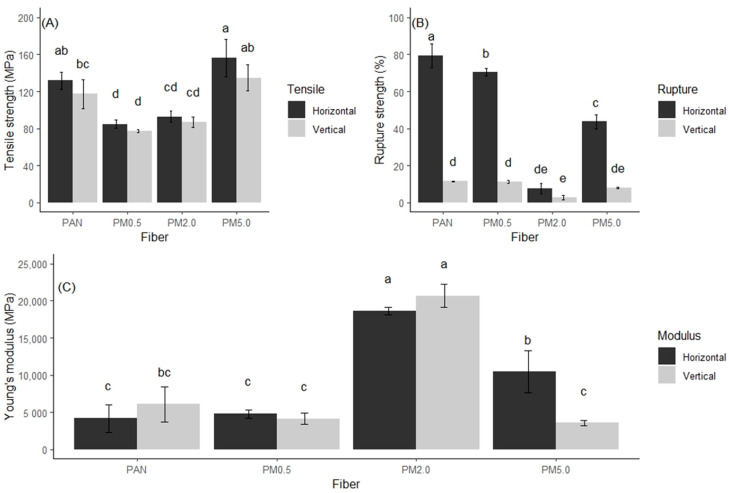
Analysis of mechanical properties of the different produced membranes, (**A**) tensile strength (MPa), (**B**) rupture strength (%), and (**C**) Young’s modulus (MPa) in PAN electrospun membranes in vertical and horizontal direction. Control (PAN), PAN + 0.5% of melanin (PM0.5), PAN + 2.0% of melanin (PM2.0), and PAN + 5.0% of melanin (PM5.0). Bars represent means ± SE (n=5) for all physical properties. Different letters indicate significant differences between all factor combinations (univariate ANOVA after post hoc Tukey test, *p* < 0.05).

The physical mechanical properties of PAN and PAN–melanin membranes (Figure 9) are significantly influenced by the addition of melanin and the orientation of the fibers. Melanin concentrations impact the differential tensile and rupture strengths and the Young’s modulus differently. For example, the higher melanin concentrations (2.0% and 5.0%) added to the fibers enhance the tensile strength in the horizontal orientation (Figure 9A). This improvement can be attributed to the reinforcing effect of melanin particles within the polymer matrix. Melanin acts as a filler, providing additional support and resistance against tensile forces applied in the horizontal direction. However, the vertical orientation shows variable effects because the alignment and distribution of fibers differ, and melanin may not provide the same level of reinforcement due to differences in stress distribution. The addition of melanin generally decreases the rupture strength (Figure 9B), with the most significant reduction observed at the 0.5% and 5.0% melanin concentrations. This reduction can be explained by the introduction of heterogeneities and potential stress concentrators within the fiber matrix. Melanin particles can create points of weakness where cracks can initiate and propagate with ease, leading to a lower rupture strength. At lower concentrations (0.5%), the melanin may not be well-distributed, leading to uneven stress distribution. At higher concentrations (5.0%), the excessive amount of melanin can disrupt the polymer network, further compromising the material’s integrity. Higher melanin concentrations (2.0%) significantly increase the stiffness of the fibers, as indicated by the increased Young’s modulus (Figure 9C). This increase in stiffness can be attributed to the rigid nature of melanin particles, which enhance the overall rigidity of the composite material. The melanin particles restrict the mobility of the polymer chains, making the material more resistant to deformation under applied stress. However, at very high concentrations (5.0%), the stiffness may not increase further or could even decrease slightly due to potential aggregation of melanin particles, which might lead to an uneven distribution and potential flaws within the matrix. The orientation of the fibers plays a crucial role in determining the mechanical properties. In the horizontal orientation, the alignment of fibers may allow for better load distribution and more effective reinforcement by melanin particles. In contrast, the vertical orientation may not benefit as much from melanin addition due to differences in how the fibers bear the applied loads and how the melanin is distributed throughout the matrix.

### 3.7. Antioxidant Activities

**Figure 10:**
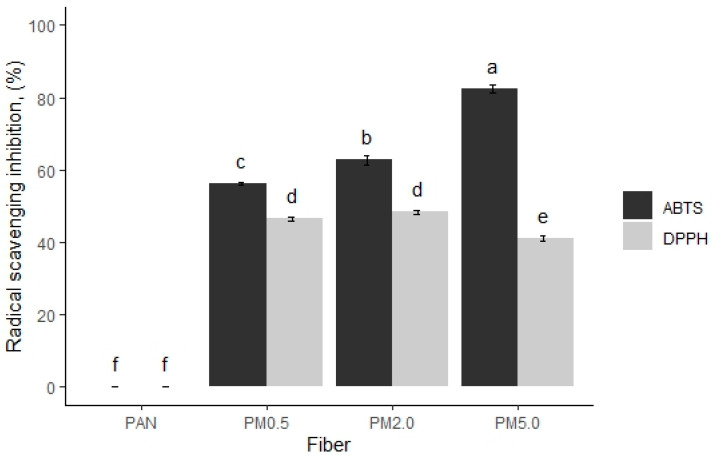
Free radical scavenging activity of melanin membranes against DPPH and ABTS. Control (PAN), PAN + 0.5% of melanin (PM0.5), PAN + 2.0% of melanin (PM2.0), and PAN + 5.0% of melanin (PM5.0). Bars represent means ± SE (n=3) for radical scavenging in %. Different letters indicate significant differences between all factor combinations (univariate ANOVA after post hoc Tukey test, *p* < 0.05).

Melanins are efficient free radical scavengers and protect different organisms against oxidative damage by neutralizing free radicals. The antioxidant capacity of fibers containing varying concentrations of melanin was evaluated against two chemical oxidants such as DPPH and ABTS Figure 10, Supplementary Figure S1).

It is possible to observe that control PAN fibers do not possess antiradical scavenging activity against the DPPH and ABTS radicals. However, fibers loaded with melanin PM0.5, PM2.0, and PM5.0 show activity with values of 56.2% ± 0.3%, 62.7% ± 1.2%, and 82.3% ± 1.0% against ABTS, and 46.5% ± 0.5%, 48.4% ± 0.5%, and 41.0% ± 0.7% against DPPH, respectively (Figure 10). Melanin’s capacity to effectively scavenge free radicals is the mechanism behind its antioxidant activity. Free radicals are extremely reactive chemicals that have the potential to destroy cells. Melanin can neutralize them. Melanin reduces oxidative stress and protects cells and tissues by retaining free radicals. Additionally, melanin can act as an extracellular redox buffer, helping to neutralize oxidants generated by environmental stress. This antioxidant mechanism contributes to melanin’s ability to protect against cellular damage and promote health in living organisms [82,83,84,85]. In that sense, the PAN with melanin demonstrated a considerable antioxidant capacity against ABTS or DPPH assays. The antioxidant capacity of the fibers loaded with melanin likely attributed to their molecular structure, in particular the different functional groups in the heterocyclic rings, o-quinones, and other reducing groups with oxidative capacity such as o-hydroquinones; therefore, melanin has a high potential to donate or capture electrons from different electronegative elements such as hydroxyl radicals, superoxide anions, singlet oxygen, or peroxyanions [86]. Melanins from different microorganisms contain a high antiradical activity against different oxidants such as DPPH and ABTS [83,87,88].

### 3.8. Antibacterial Activity

The control PAN fiber does not possess antimicrobial activity; however, the addition of any melanin concentration to PAN fibers inhibits microbial growth comparably to the positive controls such as the antibiotics gentamicin and ampicillin. Furthermore, a higher amount of melanin incorporated into the fibers produces a greater inhibitory effect [42]. Specifically, the inhibition halos against bacteria were proportional to the melanin concentration; for example, when the nanomembrane was loaded with 5% of melanin, we found halos of 7.68 mm against *S. aureus*, 7.61 mm for *E. coli*, and 8.0 mm for *P. aeruginosa* (Table 1, Supplementary Figure S2).

The inhibition results are aligned with previously reported findings, indicating that melanin exhibits antimicrobial activity against gram-positive and gram-negative pathogens such as *Bacillus pumilus*, *Staphylococcus warneri*, *Pseudomonas aeruginosa*, *Bacillus altitudinis*, *Brevibacterium casei*, *Micrococcus luteus*, *Bacillus* sp., *Geobacillus stearothermophilus* and *Streptococcus mutans*, *Escherichia coli*, *Staphylococcus aureus*, *Bacillus megaterium*, and *Salmonella* [89,90]. The presence of the reactive oxygen species (ROS) in cells has garnered interest due to their ability to decompose organic compounds and inhibit bacterial proliferation. ROS includes oxygen free radicals and any other oxygen-containing molecule in which the oxygen atom has greater reactivity than molecular oxygen (O_2_). The elevated reactivity enables ROS to extract electrons from organic compounds, leading to the degradation of these compounds into water and carbon dioxide. This similar reactivity damages the cell membrane of bacteria, which results in their death. Allomelanin derived from walnut shells contains catecholic compounds that, when oxidized, generate reactive oxygen species. The oxidation of these catecholic compounds involves electron transfer that converts O_2_ into superoxide (O_2_^−^) and hydrogen peroxide (H_2_O_2_). The release of H_2_O_2_ interacts with the cell membrane or cell wall of microorganisms, resulting in cellular destruction [4,42,91]. Finally, compounds synthesized by plants as a defense mechanism can act by interacting with the cell membrane or cell wall of microorganisms, causing changes in membrane permeability, which results in cell destruction. They are also capable of penetrating bacterial cells and promoting the coagulation of their contents [4].

### 3.9. Antiaging Activity

The evaluation of enzymatic inhibition against skin-related aging enzymes, including tyrosinase, hyaluronidase, elastase, and collagenase, was conducted to assess the efficacy of melanin-loaded nanomembranes (Table 2). This analysis was crucial in determining the potential antiaging properties of the fabricated materials. Notably, polyacrylonitrile (PAN) fibers without melanin incorporation demonstrated no significant enzymatic inhibition potential. However, the addition of melanin to the polymer mixture demonstrated effective inhibition. Additionally, we observed that all concentrations of melanin have inhibitory effects against the four enzymes (Table 2). Enzymatic inhibition is dependent on the concentration of melanin, and we observed that the membranes with 5.0% of melanin (PM5.0), inhibited the activity of tyrosinase (36%), hyaluronidase (22%), collagenase (25%), and elastase (28%). The fibers loaded with 2.0% (PM2.0) of melanin, dropped the enzymatic activities in tyrosinase (35%), hyaluronidase (15%), collagenase (25%), and elastase (28%), respectively, and the fiber with the lowest melanin concentration (PM0.5) lightly diminished the enzymatic activity for tyrosinase (0.8%), hyaluronidase (9.8%), and collagenase (10.8%) but not for elastase (27%) (Table 2). There are no reports on inhibiting antiaging enzymes using melanin-loaded fibers or the pigment alone. However, several studies have used polyphenols and peptides with high inhibition of enzymatic activities for tyrosinase, hyaluronidase, collagenase, and elastase [92,93,94,95,96]. For example, peptides derived from chia seed hydrolysates showed 38% enzymatic inhibition against collagenase, similar to our results, as well as enzymatic inhibition for elastase (43%) and tyrosinase (61%) [57]. Similarly, polyphenolic extracts from grape pomace revealed enzymatic inactivation for elastase (17–47% inhibition) and collagenase (24–43%) [45]. Our results, in conjunction with these findings, suggest promising potential for the development of cosmetic formulations. Specifically, these bioactive compound-loaded nanomembranes could serve as a foundation for innovative facial masks with enhanced antiaging properties.

### 3.10. Docking In Silico of Each Tested Enzyme with Melanin

Molecular docking results indicate that melanin exhibits a stronger binding affinity for collagenase compared to the re-docked inhibitor, as evidenced by the lower binding energy of −10.4 kcal mol^−1^ for melanin versus −9.3 kcal mol^−1^ for the inhibitor (Table 3). This suggests that melanin could potentially be a more competitive inhibitor of collagenase. Further, results show that melanin exhibits similar binding affinities for elastase and tyrosinase compared to the re-docked inhibitors. The binding energies of melanin and the re-docked inhibitors for elastase are −7.1 and −7.6 kcal mol^−1^, respectively, and for tyrosinase, they are −6.3 kcal mol^−1^ for both. These comparable binding energies suggest that melanin might have a similar inhibitory potential for these enzymes as the reference inhibitors. Distinct collagenase inhibitors, often featuring a zinc-binding group (ZBG), have been identified. These inhibitors target the catalytic zinc ion, rendering the enzyme inactive. However, their lack of selectivity over human metalloproteinases (MMPs) hinders their clinical development [58].

**Figure 11:**
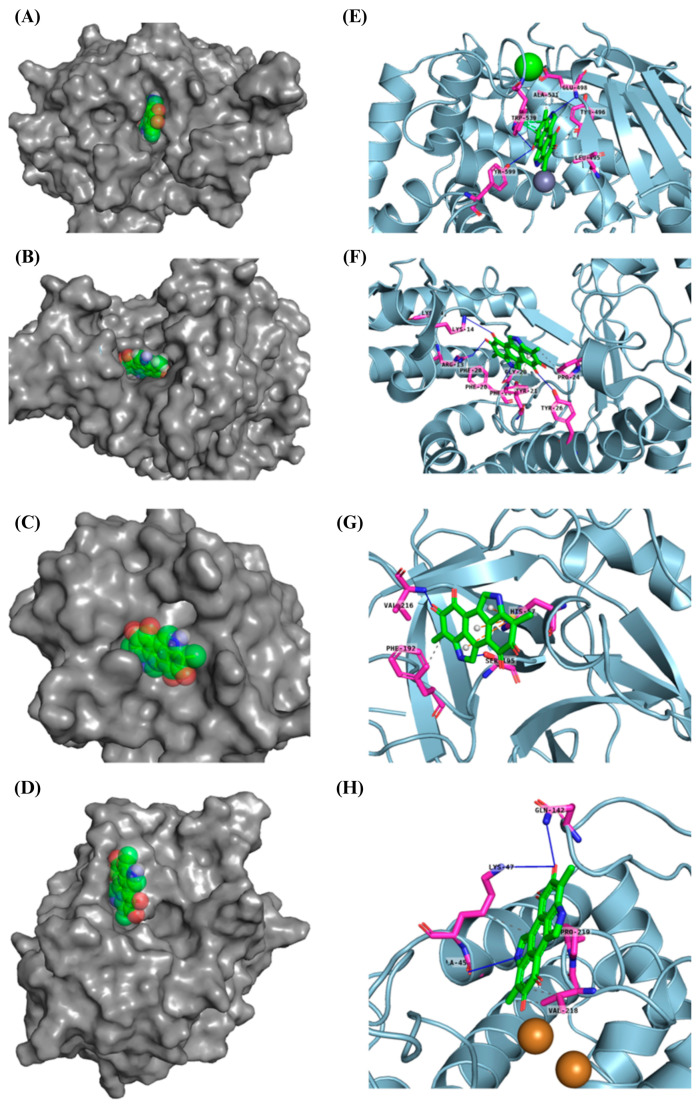
Tridimensional structure of the molecular docking between melanin pigment and the catalytic sites of various aging-related enzymes. Surface (**A**–**D**) and cartoon (**E**–**H**) representations of the predicted models illustrate interactions between melanin and the enzymes: collagenase (**A**,**E**), hyaluronidase (**B**,**F**), elastase (**C**,**G**), and tyrosinase (**D**,**H**). The surface models depict the most accurate predictions, while the cartoon representations highlight the amino acids (pink) involved in the protein–ligand interactions within the enzyme binding pocket and melanin (green). In (**E**), green and gray dots represent calcium (Ca) and zinc (Zn) atoms, respectively. Brown dots in (**H**) depict copper (Cu) atoms.

The experimental crystal structure of collagenase reveals that the ortho-acetamide group of the inhibitor IFW forms hydrogen bonds with Glu498 and engages in π-π stacking interactions with Trp539. The triazole ring interacts with Glu555 and Tyr599, while the hydroxamate group coordinates with the zinc ion. Our molecular docking results suggest that melanin interacts with key residues within the active site of the target protein, similarly to the known inhibitor IFW (Figure 11). Specifically, melanin appears to form hydrophobic interactions with Glu498 and Trp539 and hydrogen bonds with Tyr599. The crystal structure of hyaluronidase (2PE4) reveals a molecule composed of two distinct domains: a catalytic domain and a novel EGF-like domain, which is often involved in protein–protein interactions and regulatory processes [59].

While no experimentally determined crystal structures of hyaluronidase–inhibitor complexes are currently available, our computational results suggest that melanin can form a stable complex within the enzyme’s active site, with a predicted binding energy of −8.6 kcal mol^−1^. This interaction is stabilized by a combination of hydrophobic interactions, hydrogen bonds, π-π stacking, and π-cation interactions. Previous studies have demonstrated that garcinol binds to human hyaluronidase (2PE4) with a binding affinity of −4.41 kcal mol^−1^, forming interactions with key residues Leu381, Asn383, Leu379, Lys377, and His305, as well as two hydrogen bonds with Asp408 [97]. Our results indicate that melanin interacts within a binding pocket, forming specific bonds with Phe204, Pro249, and Tyr210. Additionally, conventional hydrogen bonds are observed with Gly203, Arg134, and Lys144. Computational modeling indicates that melanin can form a stable complex within the reported enzyme’s active site, stabilized by various interactions. Our inhibitory assay, along with the stronger interaction results compared to garcinol, supports melanin’s inhibitory effect. The human neutrophil elastase (HNE) used in this analysis was crystallized with a non-covalently bounded dihydropyrimidone inhibitor (DHPI) [60]. Previous studies have reported that the primary binding interactions between DHPI and elastase involve van der Waals forces with hydrophobic residues Val190, Ala213, and Val216. Additionally, π-π stacking occurs between the phenyl ring of Phe192 and the trifluoromethylphenyl group of DHPI, while Asp226 participates in hydrogen bonding networks, and Val216 appears to be a key residue for binding peptidic inhibitors [60,98]. Our results show that the molecular docking of melanin with HNE shares similar binding energies of 7.6 kcal mol^−1^ for melanin and 7.1 kcal mol^−1^ for DHPI. Furthermore, the predicted binding pose suggests interactions with key residues, like those described above, such as Phe192, Ser195, and Val216 (Table 4).

The experimental data on the binding pose of B5N in the tyrosinase active site show that the 4-fluorobenzyl moiety is oriented towards CuA and stabilized by stacking interactions with His208. Additionally, a polar interaction with Arg209 is observed; however, the flexibility of Arg209 appears to contribute to accommodating different ligands [61]. Interestingly, our results show that melanin binds to a different cavity in the protein’s binding site compared to B5N; however, the predicted binding energy is similar, at −6.3 kcal mol^−1^ for both ligands. Our melanin–tyrosinase docking analysis revealed two key interaction types: hydrophobic interactions involving Lys47, Val218, and Pro219 and hydrogen bonds formed with Gly46, Lys47, and Gln142. Overall, the computational analysis revealed similar binding modes and affinities between melanin and known inhibitors for each target protein, corroborating the experimental observations that melanin-loaded PAN nanofibers possess significant bioactive potential.

## 4. Conclusions

This study demonstrates that PAN fibers infused with melanin exhibit a dense and homogeneous structure, with the fiber diameter increasing proportionally to the melanin concentration. Based on the above results, membranes’ characteristics have a direct relationship with a higher concentration of melanin (5.0%) since membranes with a high concentration have excellent antioxidant and antimicrobial activity and a strong inhibitory activity against aging-related enzymes. The incorporation of melanin preserves the original molecular structure of the PAN fibers while enhancing their mechanical properties, particularly the tensile strength at higher melanin concentrations. Release kinetics reveal that higher melanin concentrations in PAN membranes result in a more substantial and sustained release over time, attributed to the greater availability of melanin within the fibrous matrix. The PAN–melanin fibers display significant antioxidant activity, with radical scavenging capacity increasing in proportion to melanin concentration. Furthermore, these fibers exhibit antimicrobial properties. Notably, higher melanin concentrations correlate with stronger inhibitory effects against aging-related enzymes, including tyrosinase, hyaluronidase, collagenase, and elastase. The molecular docking of proteins with the melanin ligand presented good binding energies with all aging-related enzymes. These findings highlight the potential of melanin-infused PAN fibers for applications requiring enhanced mechanical properties, controlled release, antioxidant activity, antimicrobial effects, and the inhibition of aging-related enzymatic processes.

## Figures and Tables

**Table 1:** Antimicrobial capacity of PAN membranes with melanin pigment against bacterial strains.

Fiber	Antibacterial Activity		
	Mean Diameter of Inhibition Zone (mm)		
	*E. coli*	*P. aeruginosa*	*S. aureus*
PAN	0.0 ± 0.0 ^a^	0.0 ± 0.0 ^a^	0.0 ± 0.0 ^a^
PM0.5	7.1 ± 0.4 ^b^	7.3 ± 0.1.5 ^b^	6.7 ± 0.5 ^b^
PM2.0	7.4 ± 0.5 ^b^	7.8 ± 0.7 ^bc^	6.8 ± 0.5 ^b^
PM5.0	7.6 ± 0.7 ^bc^	8.0 ± 0.8 ^bcd^	7.6 ± 0.3 ^bc^
Gentamicine 10 mg	9.8 ± 0.6 ^de^	9.3 ± 0.3 ^cde^	9.9 ± 1.4 ^de^
Ampicillin 100 mg	9.8 ± 0.1 ^de^	11.3 ± 2.0 ^e^	9.5 ± 1.8 ^cde^

Values represent means ± SE (n=3) for diameter of inhibition zone (mm). Control fiber (PAN), PAN + 0.5% of melanin (PM0.5), PAN + 2.0% of melanin (PM2.0), and PAN + 5.0% of melanin (PM5.0). Different letters indicate significant differences (univariate ANOVA after post hoc Tukey test, *p* < 0.05).

**Table 2:** Enzymatic inhibition of PAN fibers loaded with melanin against aging enzymes.

	Enzyme Inhibition (%)			
Fiber	Tyrosinase	Hyaluronidase	Collagenase	Elastase
PAN	0.0 ± 0.0 ^a^	0.0 ± 0.0 ^a^	0.0 ± 0.0 ^a^	0.0 ± 0.0 ^a^
PM0.5	0.8 ± 13.3 ^b^	9.8 ± 0.7 ^b^	10.9 ± 3.8 ^b^	27.3 ± 9.5 ^b^
PM2.0	35.2 ± 8.7 ^c^	15.3 ± 3.4 ^c^	25.3 ± 4.3 ^c^	28.2 ± 7.4 ^b^
PM5.0	36.0 ± 5.9 ^c^	21.9 ± 1.4 ^d^	37.2 ± 3.0 ^d^	33.0 ± 9.6 ^b^

Values represent means ± SE (n=3) of enzymatic inhibition in %. Control fiber (PAN), PAN + 0.5% of melanin (PM0.5), PAN + 2.0% of melanin (PM2.0), and PAN + 5.0% of melanin (PM5.0). Different letters indicate significant differences (univariate ANOVA after post hoc Tukey test, *p* < 0.05).

**Table 3:** Chemical structures and binding energies of docked melanin and re-docked experimental inhibitors against their respective target proteins, as determined by molecular docking simulations.

Ligand	PubChem CID	Binding Energy (Kcal/mol)	Target Protein	Ligand Chemical Structure
2HY	11539563	−7.6	Elastase	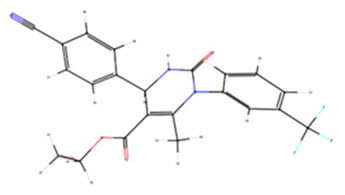
B5N	17249811	−6.3	Tyrosinase	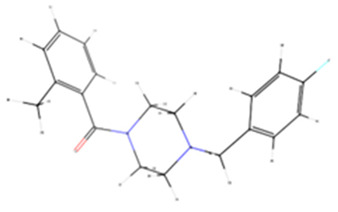
IFW	165416375	−9.3	Collagenase	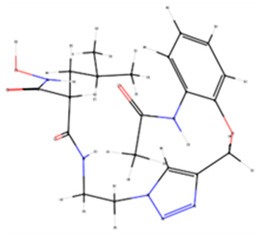
Melanin	-	−10.4	Collagenase	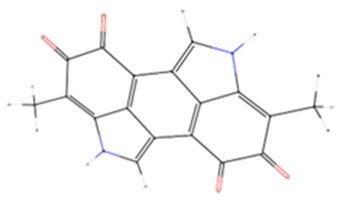
		−8.6	Hyaluronidase	
		−7.1	Elastase	
		6.3	Tyrosinase	

**Table 4:** Residue numbers and interaction types for the best binding pose of melanin with its respective target protein, as determined by molecular docking simulations.

Interaction Type	Enzyme Inhibition (%)			
	Collagenase	Hyaluronidase	Elastase	Tyrosinase
Hydrophobic	Leu495	Phe204	Phe192	Lys47
	Tyr496	Pro249		Val218
	Glu498			Pro219
	Ala531			
	Trp539			
Hydrogen Bonds	Glu498	Arg134	Ser195	Gly46
	Trp539	Lys144	Val216	Lys47
	Tyr599	Gly203		Gln142
π-π stacking π-cation	Trp539	Tyr210		Trp539

## Data Availability

The authors confirm that the data supporting the findings of this study are available within this article.

## Supplementary Materials

The following supporting information can be downloaded at https://www.mdpi.com/article/10.3390/membranes15020044/s1: Figure S1: Free radical scavenging activity of melanin membranes against DPPH and ABTS experiments; Figure S2: Antibacterial activity of membranes loaded with melanin pigment.
