# Supplementary material for: Electrospun Membranes Loaded with Melanin Derived from Pecan Nutshell (Carya illinoinensis) Residues for Skin-Care Applications
Source: Membranes (Basel). 2025 Feb 3;15(2):44. doi: 10.3390/membranes15020044 (PMC11857193; doi:10.3390/membranes15020044)
Supplement: Supplementary file 1 [file membranes-15-00044-s001.zip › membranes-3392484-supplementary.pdf]

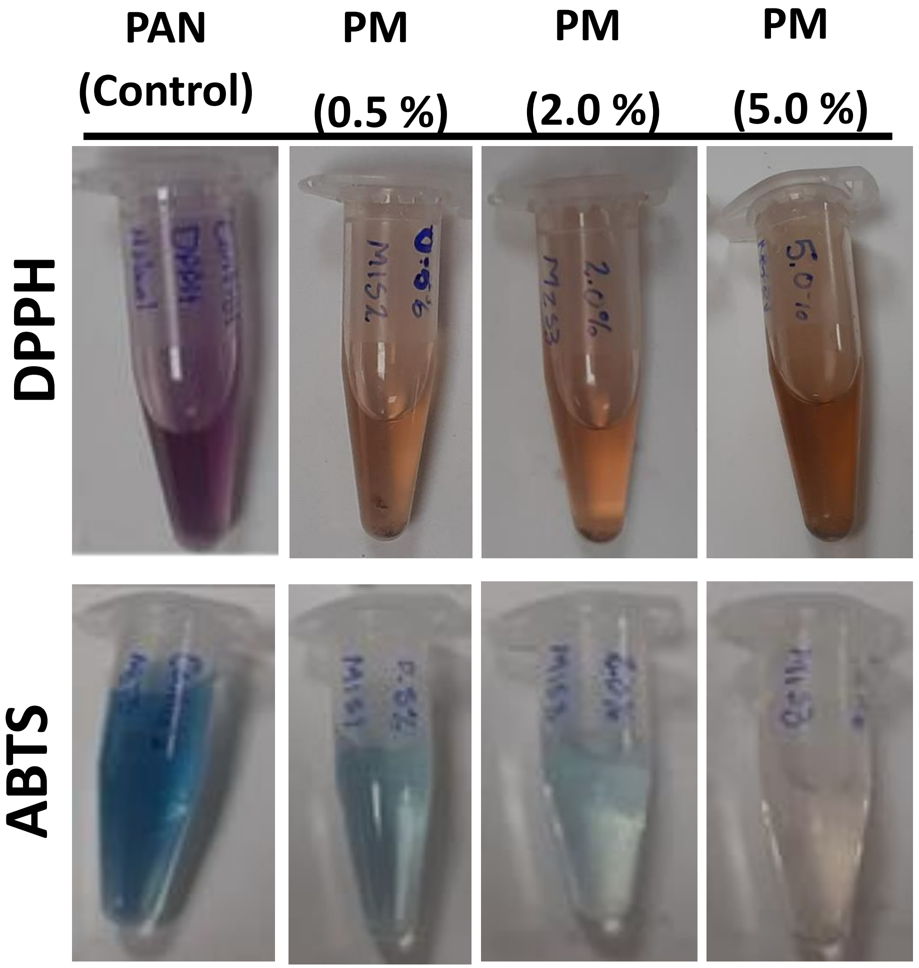

Supplementary Figure S1. Free radical scavenging activity of melanin membranes against DPPH and ABTS experiments. Control (PAN), PAN + 0.5% of melanin (PM0.5), PAN + 2.0 % of melanin (PM2.0), and PAN + 5.0% of melanin (PM5.0).

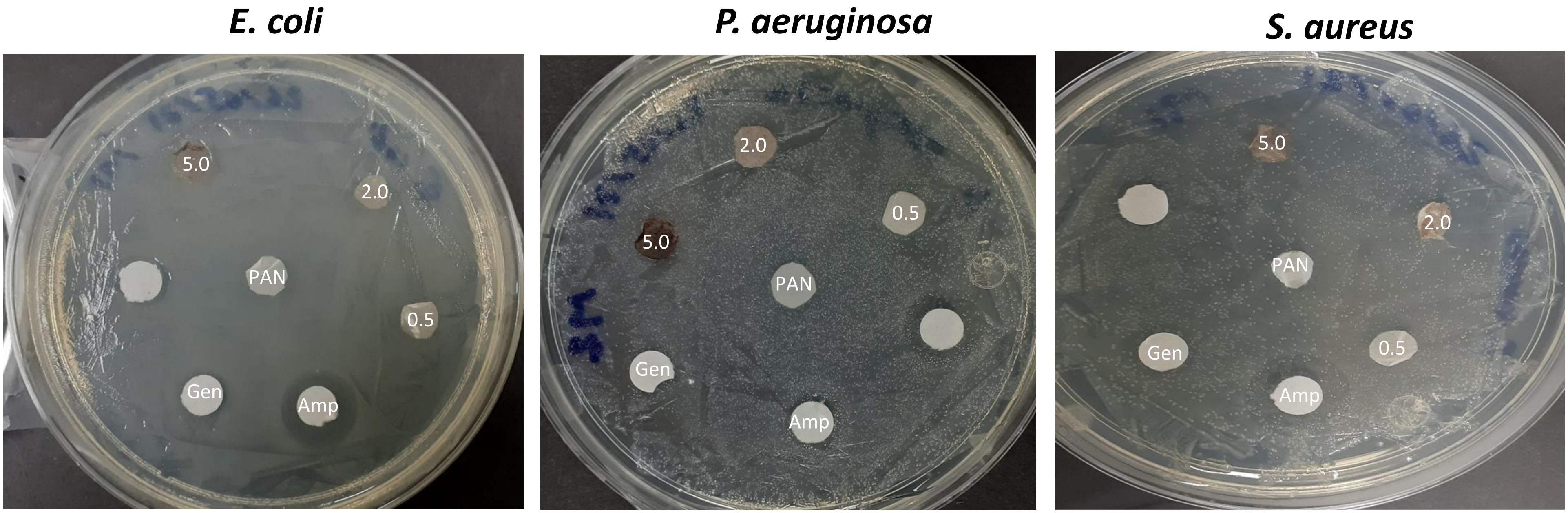

Supplementary Figure S2. Antibacterial activity of membranes loaded with melanin pigment. Control (PAN), PAN + 0.5% of melanin (PM0.5), PAN + 2.0 % of melanin (PM2.0), and PAN + 5.0% of melanin (PM5.0). Gentamincine 10 mg (Gen) and Ampicillin 100 mg (Amp).
